# Perioperative nursing in public university hospitals: an ethnography

**DOI:** 10.1186/s12912-014-0045-7

**Published:** 2014-12-09

**Authors:** Erik Elgaard Sørensen, Ida Østrup Olsen, Marianne Tewes, Lisbeth Uhrenfeldt

**Affiliations:** Department of Clinical Medicine, Aalborg University, Aalborg, Denmark; Clinical Nursing Research Unit, Aalborg University Hospital, Aalborg, Denmark; Department of Gastrointestinal Surgery, Aalborg University Hospital, Aalborg, Denmark; Heart Center, Rigshospitalet, Copenhagen, Denmark; Department of Health, Science and Technology, Aalborg University, Aalborg, Denmark

**Keywords:** Anthropology, Cultural, Ethnography, Nursing care, Perioperative nursing, Technology

## Abstract

**Background:**

In recent years, perioperative nursing has received ongoing attention as part of an interprofessional collaboration. Perioperative nursing is constantly faced with new challenges and opportunities that necessitate continual updates of nursing knowledge and technical skills. In light of the longstanding relationship between nursing and technology, it is interesting that few studies with this focus have been performed. Therefore, our research question was: What is the content of perioperative nursing and how do nurses facilitate the interaction between nursing care and technology in highly specialized operating rooms in public university hospitals?

**Methods:**

An ethnography involving participant observations and interviews was conducted during a 9-month study period. The participants comprised 24 nurses from 9 different operating wards at 2 university hospitals in different regions of Denmark.

**Results:**

Patients were addressed as either human beings or objects. Likewise, the participants’ technical skills were observed and described as either technical flair or a lack of technical skills/technophobia. The different ways in which the technical skills were handled and the different ways in which the patients were viewed contributed to the development of three levels of interaction between technology and nursing care: the interaction, declining interaction, and failing interaction levels.

**Conclusion:**

Nursing practice at the interaction level is characterized by flexibility and excellence, while practice at the declining interaction level is characterized by inflexibility and rigidity. Nursing practice at the failing interaction level is characterized by staff members working in isolation with limited collaboration with other staff members in operating rooms. Considering that the declining and failing interaction levels are characterized by inflexibility, rigidity, and isolation in nursing practice, nurses at these two levels must develop and improve their qualifications to reach a level of flexible, excellent interaction. Nurse leaders must therefore refocus their skills on proficiency in perioperative nursing.

## Background

Perioperative nursing has been explored from different angles for more than a decade [1-3]. The interaction between nursing care and technology has been discussed in particular detail [2,4]. This discussion has raised a number of questions about the tendency to view nursing in operating rooms (ORs) as invisible to the patient and as surgical interventions without nursing activities [5,6].

Perioperative nursing as an act of technology includes the knowledge and skills to work proficiently with instruments, equipment, and machinery [7-9]. Numerous advances in technology such as robots, digital displays, artificial organs, magnetic sensors, and communications technology [7,10] require that nurses also become educated in information technology (IT) [8]. In ORs, the team members work and activities are structured around the management of the “operating list” [11].

The surgical event is viewed as a social and technical process [12] that involves the entire team, including the surgical nurse, circulating nurse, anesthesia provider, surgical technician, and surgeon. Furthermore, the content of perioperative nursing is viewed as a number of activities that often occur simultaneously. The use of specific instrumentation and procedures illustrates the central nature of the actions in ORs, where the dominant goal is to conduct a successful operation for the treatment of a specific disease or injury [6] with a focus on patient safety and prevention of surgical infection [13,14].

From a patient perspective, technology can be quite frightening, despite the fact that nurses find all aspects of perioperative nursing and the large display of technology familiar [15]. According to Sweeny [8], the use of technology and related transitions can reduce human contact. A recent review highlighted the transitions that increased patients’ anxiety [16], adding importance to the implementation of individualized nursing care in the perioperative setting.

However, the involvement of such care (e.g., when using the term “advocacy”) in perioperative nursing in the OR is unclear. A descriptive study of OR nurses’ perceptions of the implications the concept “advocacy” included interrelated and overlapping themes such as protection, communication/vocalizing, doing, comfort, and caring [17]. When Westerling and Bergbom [18] evaluated effective perioperative nursing care from the patient’s perspective, they found that the value of being acknowledged as an individual carried particular importance to the patients, and that the ability of patients to share their perioperative experience with familiar nurses made the patients feel calm, safe, and secure. This perspective was first identified by Rudolfsson *et al*. [19] and later actualized by Rudolfsson [4] in their elaboration of a model illustrating the perioperative dialogue/ethos, showing that the patients felt that it was safe to hand over the responsibility to the nurse when they felt acknowledged, listened to, and met with empathy. Likewise, another study demonstrated the creation of continuity through preoperative dialogue, its manifestation in intraoperative dialogue, and its closure in postoperative dialogue [20]. Although the interaction between nursing care and technology has been taken for granted by many OR nurses [6], the interaction has also been viewed as a challenge [21-24] because perioperative nursing has been, and remains, inextricably linked to the development of technology with the risk of eroding the quality of care [6]. Therefore, perioperative nursing is constantly faced with new challenges and opportunities that necessitate continual updates of nursing knowledge and technical skills [3,25]. In light of the longstanding relationship between nursing and technology, it is interesting that only few studies with this focus have been performed [21-24]. Furthermore, these studies are >10 years old.

With this background, the purpose of the present study was to investigate the actual content of perioperative nursing in highly specialized ORs in public university hospitals. The research question was: What is the content of perioperative nursing and how do nurses facilitate interactions between nursing care and technology in highly specialized ORs in public university hospitals?

## Methods

This ethnography was based on participant observations and interviews inspired by practical ethnographic principles [26-28]. We directly observed OR nurses in the field and interviewed them about their experience to capture the concrete, everyday practice in ORs and understand the content of perioperative nursing. The participants comprised 24 registered female nurses selected from 8 surgical specialties at 3 urban geographical locations in 2 public university hospitals to ensure diverse and nuanced data. The surgical specialties included orthopedic, thoracic, gastrointestinal, breast, ear-nose-throat, neurosurgical, urological, and gynecological surgery. The participants’ ages ranged from 31 to 63 years. They had from 3 to 24 years of nursing experience in ORs. A minimum of 3 years of clinical nursing experience was required for enrollment in this study to ensure a strong nursing identity and clinical knowledge [29,30].

Data were collected through field observations of each participant for a period of 3 to 5 days for 5 to 8 hours per day depending on the participant’s daily work in the OR. This time period was based on both experience from another field study and the aim of achieving empirical saturation [31]. This saturation was achieved when the nuances in the nurses’ experiences decreased though changes in settings and geography.

During the field observations, the authors (E.E.S. and I.Ø.O.) produced handwritten field notes [27]. Each participant observation was followed by an ethnographically inspired interview [26]. The interviews were based on semistructured interview guides individually created based on the previous field observations. One interview question was: “You told me you had technical flair. Could you please tell me more about this flair?” The interviews were intended to contribute to a deeper understanding of the context-bound events from the participant observations [28]. The overall study period included 122 operations performed during 9 months, amounting to 273 hours in 44 days and 6 nights. This strategy allowed for repetitions over time and set aside the “tip-of-the-iceberg” assumption [27].

### Data analysis and preunderstanding

The authors (E.E.S. and I.Ø.O.) transcribed all field observations, notes, and interviews into verbatim text. This text was later subjected to a hermeneutic back-and-forth process [32] in a stabilization analysis phase and an adaptation analysis phase (E.E.S., I.Ø.O., and L.U.) according to Hammersley and Atkinson’s guidelines [28:333–367].

The stabilization phase involved preparation of data for analysis, systematization, and pattern identification using the qualitative analysis program NVivo9 [33] to develop the content of perioperative nursing (first part of the research question). This led to the formation of two themes: “Technical skills” and “Understanding of the individual patient.” These themes were developed into subthemes (Table 1), and the themes and their subthemes were grouped within the first main finding: *OR nurses*’ *interaction between skills and understanding*.

**Table 1:** **Themes and subthemes of the main finding: OR nurses’ interaction between skills and understanding**

**OR nurses’ interaction between skills and understanding**	
Themes	Sub-themes
Technical skills	Technical flair
	Technically unskilled/technophobia
Understanding of the individual patient	Patient viewed as a human being
	Patient viewed as an object

In the adaptation phase, the analysis focused on gaining an understanding of the interaction between nursing care and technology in perioperative nursing (second part of the research question). Themes and subthemes of nursing care and technology were coded and collected based on mutual links and internal relationships and structures [28:241]. The coherence between the themes and subthemes contributed to the development of three levels of interaction: the “interaction level,” “declining interaction level,” and “failing interaction level” (Table 2). These three levels were grouped in the second main finding: *OR nurses*’ *interaction between nursing care and technology*.

**Table 2:** **Levels and coherence between themes and subthemes in the main finding: OR nurses’ interaction between nursing care and technology**

**OR nurses’ interaction between nursing care and technology**	
Levels of interaction	Coherence between the first main finding, the themes and sub-themes
Interaction level	Interaction between technical flair and patient viewed as a human being
Interaction declined level	Interaction between technical flair and the patient viewed as an object
	Interaction between technical unskilled and patient viewed as a human being
Interaction failed level	Interaction between technical unskilled and patient viewed as an object

Recent theory-, experience-, and research-based work [31,34,35] from public urban university hospitals with a practice-theory termed “interactional nursing practice” [36,37] has inspired our data analysis with a theoretical preunderstanding during all steps in the data analysis. The theoretical and practice perspectives of this practice theory are closely interwoven. This preunderstanding challenged and problematized the normative nature of perioperative nursing during the field observations and interviews [37]. Therefore, the observations and interviews allowed for investigation of three possible modes of action. The first is the *cognitive*-*instrumental mode of action*, which contributes to problem-solving and result-oriented activity representing technical activities. The *aesthetic*-*expressive mode of action* concerned nurses’ self-knowledge and understanding of the individual patient’s situation based on dialogue and communication. The third mode of action, the *moral*-*practical mode of action*, handles discussions and actions in relation to the patient’s overall situation. These three different modes of action were only separated for theoretical reasons in the present study; in nursing care, they are part of a whole. Their separation in nursing practice may result in narrow-minded moralism and dogmatism [36,37].

### Ethical considerations

The North Denmark Regional Research council approved the study protocol before study start (Data Protection Agency, journal no. 2008-58-0028). The study adhered to the ethical guidelines for nursing research in the Nordic countries with regard to participant information, including declarations of consent and anonymity [38]. The nursing directors at the two university hospitals were gatekeepers [28: 49]. The leaders, OR nurses, anesthesia providers, and surgeons met the researchers (E.E.S. and I.Ø.O.) during the information sessions and were informed about the investigation. Knowledge transfer was secured by a Danish publication [35]. All 24 participants provided written informed consent, and none withdrew during the study. Direct encounters between the researchers and awake patients were avoided by standing behind apparatuses or screens. When this was impossible, the researchers introduced themselves to the patients.

## Results

The results of the analysis led to two main findings. The first main finding concerned *OR nurses*’ *interaction between skills and understanding*, and the second concerned *OR nurses*’ *interaction between nursing care and technology*. In the Results section, each citation of an observation or interview statement has been assigned a reference number for one of the 24 participants, who practiced as either the circulating nurse (CN) or surgical nurse (SN).

### OR nurses’ interaction between skills and understanding

The first theme is entitled “technical skills” and concerns the different ways in which the technical assignments and developments were handled. The second theme is entitled “understanding of the individual patient” and concerns the different ways in which the patients were viewed. Table 1 shows how the relationships between the main finding (interaction between skills and understanding) and the subthemes were developed.

#### Technical skills

Technical skills were expressed in two ways: technical flair and a lack of technical skills.

Technical flair was considered to be present when nurses demonstrated skill in carrying out procedures and operating instruments regardless of the amount, size, construction, or variety of different types of machinery and equipment. Technical flair also involved the ability to easily acquire new knowledge and skills in using instruments and machinery, including the consequences of their use in a specific patient situation. Technical skills were reflected in the following observation: *The SN* [*participant*] *unpacks and prepares for the operation and says to the CN*, “*Look at the scope*—*it has been assembled before autoclaving. It is not supposed to be assembled. It has to be separated in its many parts*, *springs*, *and screws before being autoclaved*, *and then the parts have to be assembled during preparation for the operation*.” *The SN acquires a new scope*, *easily assembles it*, *and says*, “*I think I am what you would call practical. I have a flair for technical things and electronics. It is easy for me and it interests me a lot*” (21).

A lack of technical skill was characterized by the ability to assist in routine operations and apply already-known instruments, equipment, and machinery without problems. However, such nurses were unable to acquire knowledge and skills in using new instruments or establish routines involving complex technical procedures and computer-based equipment. This lack of skill had negative consequences as shown by the following interview response: “*I don*’*t have technical flair. It is not easy for me and it doesn*’*t interest me. I fall short as soon as I have to work with a computer. One day*, *I made a mistake when a patient was connected to the navigation system* [*complex computer*-*based surgical equipment*]. *I touched* ‘*something*’ *and the patient had to undergo the surgery without the advanced technique*” (1).

Technophobia was also viewed as a lack of technical skills. Technophobia was characterized by a lack of skills in certain procedures, potentially leaving the nurse feeling fearful and clumsy. This was expressed in the following statement during an operation: “*I don*’*t feel good about mixing the cement. I am afraid that I might screw things up. That* [*the procedure*] *takes up so much* [*energy*]. *It is annoying to think about during the whole operation. I feel like a clown*” (17).

#### Understanding of the individual patient

The nurses’ understanding of the individual patient was expressed in two different ways: the patient was viewed as either a human being or an object. Viewing the patient as a human being was shown by the way the nurses considered a patient’s situation during an operation: *A 65*-*year*-*old woman is lying on the operating table after an ostomy operation. The surgeon has left the operating room. When removing the sterile cover*, *the CN* [*participant*] *notices that the ostomy is* “*uneven*.” *She encourages the SN to contact the surgeon. The surgeon agrees that the ostomy does not have the desired shape. He cuts the lowest suture*, *pulls the bowel further up*, *and places a new suture. Everyone is satisfied with the shape of the ostomy* (23).

This participant was asked to elaborate on the above-described episode and said, “*It is a trauma in itself to have a life*-*threatening disease and an ostomy. If the patient*, *on top of this*, *will have problems adhering the plate and the ostomy bag because we haven*’*t done our job properly*…*well*, *that just aggravates the situation. I have worked with ostomy patients and I could see that the ostomy was not okay*” (23). In this episode, the CN applied her experience-based knowledge for the benefit of the patient. She expressed her understanding of the patient as an ill and vulnerable human being.

There were also nurses who viewed patients as objects. This was observed in a situation in which the SN did not allow the CN to speak up for a patient: *A 45*-*year*-*old woman with cancer is undergoing surgery for a pathological fracture. The CN notices that the surgeon is uncertain about the instrumentation used to measure the size of the prosthesis. The surgeon asks the SN for a prosthesis of a certain size. The CN reacts by saying*, “*But your measurement was larger than this*” [*implying that the surgeon asked for the incorrect prosthesis*]. *The SN reacts by saying to the CN*, “*Hey*!” [*implying that she was interfering with something with which she was not supposed to interfere*] (17). The prosthesis was subsequently discovered to be too large, and the patient required further surgery. According to the field notes, the SN was struck by passivity. Moreover, she prevented the CN from getting involved in the situation. In this example, the approach was characterized by a lack of interest in the patient as a human being; the patient was instead seen as an object.

### OR nurses’ interaction between nursing care and technology

In the second main finding, *OR nurses*’ *interaction between nursing care and technology*, the coherence between the themes and subthemes contributed to the development of three levels of interaction: the interaction, declining interaction, and failing interaction levels (Table 2). The different ways in which the technical assignments and developments were handled and the different ways in which the patients were viewed contributed to the development of all three levels of interaction.

#### Interaction level

The interaction level was characterized by the interaction between the presence of technical flair and viewing the patient as a human being. This was expressed in the following way: *The SN* [*participant*] *is assisting during an operation of a 72*-*year*-*old woman. The SN says*, “*I haven*’*t done this* [*specific operation*] *for a long time*.” *The SN gets five large boxes and separates instruments from a depot*, *takes the instruments to the operating room*, *and unpacks and prepares the instruments for the operation. She now places her hand on the patient*’*s shoulder after the patient has been sedated and assists in connecting her to the respirator. Later during the operation*, *the SN says*, “*She* [*the patient*] *is such a fine little lady*.” *With eyes on the surgical field and without speaking*, *the surgeon reaches a hand toward the SN*, *who passes a specific type of suture to the surgeon. The SN looks at the surgical field and says*, “*Wait. Is that the right suture for that place*? *If not*, *you*’*ll need a different one*.” *She then passes another type of suture to the surgeon* (23).

In this episode, technical flair was evidenced by the SN’s confidence in using the equipment and proficient grasp of the situation despite the fact that it had been a while since she had assisted in this type of operation. The underlying understanding of the patient as a human being was expressed during the SN’s participation in the patient’s sedation, in which she placed her hand on the patient’s shoulder while assisting the anesthetic nurse. The SN referred to the patient in a respectful manner by using the expression “such a fine little lady,” and it is evident that she understands, sees, and meets the patient as a human being.

#### Declining interaction level

The declining interaction level was characterized in two ways: as an interaction between the presence of technical flair and viewing the patient as an object and as an interaction between a lack of technical skills and viewing the patient as a human being.

The interaction between the presence of technical flair and viewing the patient as an object was expressed when a participant spoke about a colleague: “*She* [*a colleague*] *is technically very skilled. She can manage everything when it comes to technology and IT systems. Therefore*, *she is our expert*, *but only when it comes to technique. She has no interest in the patients. She cannot talk to* [*understand*] *them* [*the patients*]” (4).

The participant who spoke about the colleague was later asked to elaborate on this statement and answered, “*Yes*, *you have a point there*” (3). This colleague was perceived as a skilled technician with technical flair. Her lack of interest in vulnerable patients, however, is an example of viewing the patient as an object.

The interaction between a lack of technical skills and viewing the patient viewed as a human being is shown in the following scenario: *The SN* [*participant*] *is about to assist in a very complicated operation. She says*, “*I haven*’*t assisted in such an operation in 100 years*.” *The CN assists the SN with the preparation. After unpacking the equipment for the operation*, *the CN is about to leave the room. Very promptly*, *the SN says*, “*No*, *you can*’*t go*.” *The SN was later asked to elaborate on this episode and stated*, “*When I am insecure about the techniques*, *I get very affected by the way the surgeon enters the room and whether I can sense that he seems insecure. Today*, *when we were using new equipment*, *there had to be a technically minded colleague next to the surgeon to assist him. And while my colleague is technically minded*, *I am caring*-*minded. I am very considerate of the sedated and defenseless patient. I see him as a human being*” (4). In this example, the technically unskilled nurse was insecure and using new equipment. The nurse acknowledges that she was insecure and expressed the need to have a technically skilled nurse present in the OR. The expressions “*No*, *you can*’*t go*” and “*I get very affected*” reflect the presence of technophobia in this technically unskilled nurse. In this scenario, it seemed as though the lack of technical skills was legitimized by viewing the patient as a human being.

#### Failing interaction level

The failing interaction level was characterized by the interaction between a lack of technical skills and viewing the patient as an object. This was demonstrated in a scenario involving the above-described 45-year-old woman with cancer who underwent surgery for a pathological fracture (see earlier theme, “Understanding of the individual patient”). In contrast to the CN, the SN did not interfere with the surgeon and his novice use of the instrumentation while measuring the prosthesis size. Furthermore, the SN prevented her colleague from providing the novice surgeon with important knowledge by saying, “*Hey*!” [*implying that she was not supposed to interfere*] (17). At the end of the operation, the surgeon said to the SN, “*I have not been satisfied with your assistance*.” The SN replied, “*Well*, *it is not my fault that you chose a prosthesis that was too big. You are supposed to know how this should be done*” (17). Before the operation, this particular participant said, “*I don*’*t bother about the patient contact. I have often felt that I am unable to do anything for them* [*patients*]” (17). According to the field notes, the SN exhibited passivity and was unable to share her (limited) technical, practical, and experience-based knowledge. Moreover, she prevented the CN from getting involved in the situation. In this example, the lack of technical skill was combined with a lack of interest in the patient as a human being; the patient was only an object.

## Discussion

The purpose of this descriptive study was to investigate the content of perioperative nursing in highly specialized ORs in public university hospitals and elucidate perioperative nurses’ interactions between nursing care and technology. The findings suggest three different levels in which perioperative nurses navigate between nursing care and technology. Thus, this study supports earlier research regarding nurses’ ability to combine technical and relational skills [6,21-24]. Bull and FitzGerald [6] reported similar findings from an ethnographic study in Australia. They stated that the necessity of combining technological proficiency and caring in the OR was taken for granted by nurses. However, our study suggests that this is not always the case when the interaction between nursing care and technology is declining or failing.

Research-based knowledge is needed to inform leaders and nurses about the technological and nursing tasks involved in perioperative nursing [39,40] and to apply this knowledge to patient safety [16]. According to Scheel [36], the cognitive-instrumental mode of action in this study is presented by the nurses’ different levels of technological skills, which range from technical flair to a lack of technical skills. The aesthetic-expressive mode of action is observed by the way Informant 23 referred to the patient in a respectful manner. The opposite occurred when Informant 17 stated that she did not care about patient contact. The third mode of action, the moral-practical mode, is represented by Informant 23, who applied her experiential knowledge about ostomy care. This was what the patient and the actual situation demanded from the nurse. The opposite occurred when Informant 17 prevented a colleague from speaking up for the benefit of the patient.

These three different modes of action require unification [36]. At the *interaction level*, the interactions among the three modes of action are always part of the current patient–nurse interaction. At the *declining interaction level*, the cognitive-instrumental or aesthetic-expressive mode of action is particularly prioritized depending on the individual nurse. Thus, the three modes of action do not always interact at this level. This leads to the risk of inadequate nursing care unless the OR is staffed with nurses with different skills who can ensure an interaction among the three modes of action. The *failing interaction level* was characterized by a lack of interaction among the three modes of action. According to Scheel’s [36] terminology, this is not nursing. This interpretation was confirmed by Bull and FitzGerald [6] who concluded that the combination of technological proficiency and patient-focused ethics of care defines whether nurses’ actions in the OR can be characterized as “nursing” (*interaction level*) rather than “technical” tasks (*failing interaction level*). In other words, there is a risk that technology undermines care. However, the present study also showed that nurses at the *declining interaction level* prioritize nursing care, perhaps because of their lack of technical flair. Interestingly, however, most studies on perioperative nursing have focused on nursing care in contrast to OR nurses’ technical skills [2,4,41,42]. Barnard [21] and Sandelowski [23] are well known for their theoretical studies on nursing and technology. Similarly, Barnard and Gerber (1999) investigated nurses’ understanding of technology through interviews. Nevertheless, the study was based on ethnographic principles and thus revealed new dimensions in technical skills. Some nurses possessed technical flair, while others were technically unskilled. This raises new questions and concerns about nurses’ varying technical skills and may thus inspire further research and discussion.

In this study, nurses at the *interaction level* combined nursing care and technology with self-reflection and a great understanding of the interdisciplinary team. Nursing practice was characterized by flexibility and excellence because the same nurse interacted with all three modes of action [37] in that she managed all tasks in the OR. Nurses at the *declining interaction level* prioritized either the technical or the nursing care dimension, and self-reflection was directed toward elements of the tasks in the OR. A match between two different nurses in the OR is required to ensure interaction among all three modes of action. This limits the implementation of perioperative nursing because the nurses cannot perform all of the tasks in the OR. Nurses at the *failing interaction level* lacked self-reflection and showed no interest in the patient. New technological challenges were limited by the nurses’ lack of technical skills. Nursing practice was characterized by inflexibility and rigidity because the nurses worked in isolation with limited collaboration with the other staff members. This interpretation was confirmed by Coe and Gould [43] and Finn [44], who claimed that well-functioning interdisciplinary teamwork is described as excellent. Thus, the present study’s findings regarding flexible versus inflexible nursing practice add to the discussion on generalist versus specialist nurses [45], as well as to the discussion on seeing the big picture in nursing, which indicates a desire to provide good care to both patients and staff [46]. An individual who fails to see the big picture might act rigidly, rather than appropriately, resulting in blind action due to mechanical and automatic thinking.

The findings in this study suggest three different levels at which perioperative nursing care and technology interact in highly specialized ORs in public university hospitals. This categorization of perioperative nursing into levels is not new. In a quantitative, descriptive, correlational study of perioperative nurses’ ability to think critically, Fesler-Birch [3] calculated the average level of critical thinking to be 2.12 on a scale of 1 to 4, in which 1 indicates no critical thinking and 4 indicates complex critical thinking. Because critical thinking may be central to nurses’ ability to meet patients’ expectations regarding care and skill, this average level of 2.12 can be costly from a patient perspective, in that as intraoperative problems arise, quick clinical judgment decision making may weaken. Fesler-Birch’s study cannot be compared to the present study in that the two were based on different methodologies. However, a number of new questions are raised when comparing one study to the other. For example, could a correlation exist between perioperative nursing at the failing interaction level, which is characterized by nurses’ lack of self-reflection, and level 1 critical thinking, which is characterized by the absence of critical thinking? On the contrary, could a correlation exist between perioperative nursing at the interaction level, which is characterized by nurses’ self-reflection, and level 4 critical thinking, which is characterized as complex? If so, both the failing interaction level and level 1 critical thinking can be costly from a patient perspective. Although there is no solid basis for this conclusion, these questions may inspire further research and discussion.

### Limitations of this study

In this study, the content of perioperative nursing was analyzed based on data from highly specialized ORs in two public university hospitals, and the results were interpreted as main findings, themes, and subthemes. Because of the particular sample and special health care context, the findings may be dismissed as unique with no scientific value. However, there are aspects of the universal within the unique [47,48]. Accordingly, the main findings of this study might be applicable by perioperative nurses at other hospitals. A few limitations are noteworthy. For example, the fieldwork was performed in daytime and in the evening, and the night hours were limited. Therefore, the study did not address the content of perioperative nursing and what characterizes this practice in relation to emergency surgery in night hours The study context focused on perioperative nursing in highly specialized ORs in public university hospitals, which are characteristically populated by seriously ill and vulnerable patients undergoing complex surgical procedures. Because the study context did not focus on the research question as related to less severely ill patients and short-term surgical procedures, this should also be seen as a limitation.

Field relationships between informants and researchers are central to any ethnographic study [49]. A typical question raised is how to account for bias associated with the fact that the participants knew that they were being observed. According to Hammersley and Atkinson [28], the underlying belief is that human behavior cannot be studied in isolation or independently from the context in which it occurs. Contextualizing the data enables the researcher to place it within a broader perspective and capture a more holistic view. This involves extensive fieldwork in naturalistic settings for prolonged time periods in which the researcher has direct, personal, face-to-face contact with informants [49]. In the present study, we observed individual nurses for 3 to 5 days for 5 to 8 hours per day. Pretending to be a completely different person than who you are is impossible for such a long period of time [34]. Therefore, we were able to capture more than just a snapshot of the nurses’ activity and were able to observe routine, repeated, and patterned social practices and processes.

## Conclusions

Perioperative nursing in highly specialized ORs in public university hospitals is performed at three different levels depending on the interaction between nursing care and technology. This leads to different characteristics of practice: Practice at the *interaction level* is characterized by flexibility and excellence because nurses exhibit interaction among (a) technological activities based on technical flair, (b) an understanding of the individual patient and self-reflection based on dialogue and communication, and (c) acting in relation to the patients’ overall situation. Nurses at the *interaction level* perform all tasks in the OR. Practice at the *declining interaction level* is characterized by less flexibility because nurses prioritize either (a) technological and instrumental activities based on technical flair or (b) nursing care based on an understanding of the individual patient. Nurses at the *declining interaction level* are unable to perform all tasks in the OR. Finally, practice at the *failing interaction level* is characterized by inflexibility and rigor because nurses’ self-reflection and interest in the patient are lacking. New technological challenges are limited by nurses’ lack of technical skills or technophobia. Nurses at the *failing interaction level* work in isolation with limited collaboration with other staff in the OR.

The findings of this study are useful in organizational, clinical, and educational settings in updating policies for perioperative nursing and enlarging perioperative nurses’ understanding of the relationship between nursing care and technology. Considering that practice at the *declining interaction level* and *failing interaction level* is characterized by inflexibility and isolation, nurses at these two levels must develop their competency to the flexible and excellent *interaction level*. An important task for nurse leaders with respect to recruiting and retention is to be aware of the need for proficiency in this field. The present findings also have some potential in relation to other areas where technology is increasing in the nursing field; e.g., the use of telehealth and technology to support older people in their homes as well as other highly technical areas such as high-dependency units and intensive (coronary) care.

Our discussion is an example of how to use nursing theory in research and expressions such as nursing care and technology, which might lead to constrained nursing practices if misunderstood. Further empirical studies are required to challenge our conclusion that nurses perform perioperative nursing in highly specialized operating departments at three different levels depending on the interaction between nursing care and technology.
